# Mapping quantitative traits and strategies to find quantitative trait genes

**DOI:** 10.1016/j.ymeth.2010.07.007

**Published:** 2011-02

**Authors:** Jonathan Flint

**Affiliations:** Wellcome Trust Centre for Human Genetics, Roosevelt Drive, Oxford OX3 7BN, UK

## Abstract

In 1999 a meeting took place at the Jackson Laboratory, a large mouse research centre in Bar Harbor, Maine, to consider the value of systematically collecting phenotypes on inbred strains of mice (Paigen and Eppig (2000) [1]). The group concluded that cataloguing the extensive phenotypic diversity present among laboratory mice, and in particular providing the research community with data from cohorts of animals, phenotyped according to standardized protocols, was essential if we were to take advantage of the possibilities of mouse genetics. Beginning with the collection of basic physiological, biochemical and behavioral data on nine commonly used inbred strains, the project has expanded so that by the beginning of 2010 data for 178 strains had been collected, with 105 phenotype projects yielding over 2000 different measurements (Bogue et al. (2007) [2].

## 1 Introduction

In 1999 a meeting took place at the Jackson Laboratory, a large mouse research centre in Bar Harbor, Maine, to consider the value of systematically collecting phenotypes on inbred strains of mice [1]. The group concluded that cataloguing the extensive phenotypic diversity present among laboratory mice, and in particular providing the research community with data from cohorts of animals, phenotyped according to standardized protocols, was essential if we were to take advantage of the possibilities of mouse genetics. Beginning with the collection of basic physiological, biochemical and behavioral data on nine commonly used inbred strains, the project has expanded so that by the beginning of 2010 data for 178 strains had been collected, with 105 phenotype projects yielding over 2000 different measurements [2].

**Fig. 1:**
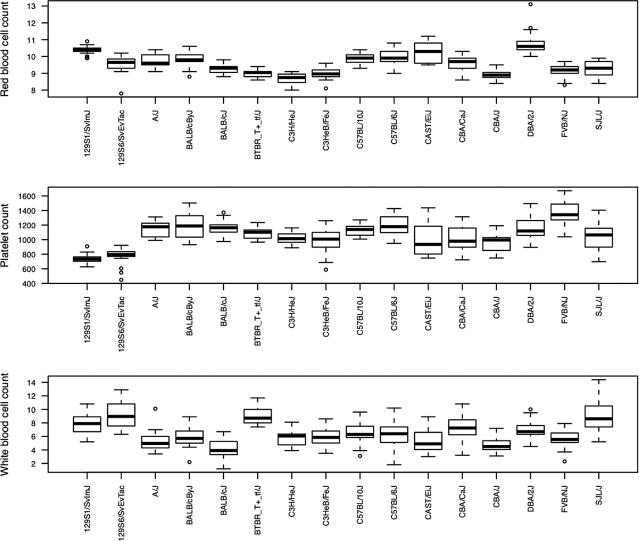
The distribution of three phenotypes in 16 inbred strains of mice shown as box and whisker plots. The *y* axis label is the name of the phenotype; the *x* axis labels are the strain names. Phenotypic data are from http://www.phenome.jax.org/pub-cgi/phenome/mpdcgi?rtn=docs/aboutmpd.

Fig. 1 provides a representative example, in this case data for 16 strains for three haematological phenotypes (red blood cell, platelet and white blood cell counts). The distribution of each strain phenotype is shown as a box and whisker plot, from which it is immediately clear that there is large and highly significant variation between strains (for example an analysis of variance for the red blood cell phenotype gives an *F* value of 56.1 (on 15 and 381 degrees of freedom), with a *P*-value of <10^−85^). Similar plots can be made for the other phenotypes, all illustrating the large differences that exist between laboratory strains.

Under the reasonable (but not entirely true) assumption that animals of the same strain are genetically completely identical then the phenotypic variation within a strain is due to environmental effects. Variance between strains provides a measure of the extent to which genetic variation contributes to phenotypic variation. So an estimate of the heritability of a trait can be obtained as a ratio of variation between strains to the total variation (between and within strain variance). For the red blood cell phenotypes this gives a heritability of about 70% – in other words, about 70% of the variation in red blood cell counts between inbred strains arise from the sequence variants that distinguish strains. While the extent of heritability varies considerably between phenotypes, it is rare to find instances where it less than 10%. Genetic effects on phenotypic variation are therefore almost always important. This observation means that genetic analysis is an entry point to the study of almost any phenotype you can measure in mice. However, as this article sets out to show, progressing from phenotype to sequence variant is a much more difficult procedure than demonstrating that the phenotype has a heritable component.

## 2 Genetic mapping

There are two factors that have shaped approaches to mapping the genetic basis of complex traits in mice: first, the existence of so many inbred strains, and second the fact that most inbred strains are related. The number and genetic consistency of inbred strains, carefully maintained to reduce genetic contamination, provides an unusual, almost unique, opportunities for genetic mapping. The relatedness between strains however can complicate some of the strategies employed. Below I consider these factors and their relationship to genetic mapping methodologies.

The utility of inbred strains for genetic mapping is best illustrated through the use of mice that contain one portion of their genome from one strain, and the rest from a second strain. The aim here, as elsewhere in this article is to find which sequence variants contribute to the differences between strains (this is by no means the only object of genetic mapping, but I do not consider here the many alternatives).

## 3 Chromosome substitution strains

**Fig. 2:**
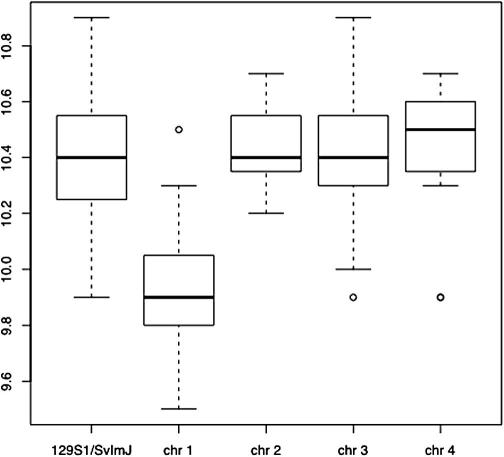
A simulated example of the distribution of red blood cell counts in four consomic strains compared to 129S1/SvlmJ. The chromosomal structure of the consomic strains is indicated by their name so “chr 1” means that all the chromosomes are derived from 129S1/SvlmJ and chromosome 1 from the other progenitor, C3HeB/FeJ.

We know from phenotype comparisons described above that strains 129S1/SvlmJ and C3HeB/FeJ contain functional genetic variants that contribute to differences in red blood cell counts, but we do not know how many there are or where they are in the genome. Suppose we create mice that have one chromosome from strain C3HeB/FeJ, and the remainder from strain 129S1/SvlmJ. Such strains are called chromosome substitution strains, for obvious reasons, or consomic (for less obvious reasons). A full consomic panel for mapping consists in mice of 22 strains (19 autosomes, 2 sex chromosomes and mitochondria), or 44 if a reciprocal set (with both parents as progenitor donor) is used. Fig. 2 shows an example of what might be found from comparing the phenotypes of four such new inbred strains with 129S1/SvlmJ (the data are again the red blood cell count whose provenance is described above). In this case the strain that has chromosome 1 from C3HeB/FeJ (in the figure shown as “chr 1”) clearly has a low RBC phenotype. Since the difference between 129S1/SVlmJ and the chromosome 1 consomic is the C3HeB/FeJ sequence on chromosome 1, we can conclude that there must be one, or more, loci that contribute to the strain difference in red blood counts on chromosome 1. Since the trait is quantitative, the locus is referred to as a quantitative trait locus or QTL. Analysis of chromosome substitution strains (CSS) is a simple way of mapping QTL.

CSS have a long history in plant [3] and Drosophila genetics [4] but have only been recently available to the mouse community. CSS were first used to map QTL in mice in 1999 [5], theoretical aspects were described in 2000 [6] and the first complete CSS set, created from A/J and C57BL/6J strains, was produced in 2004 and used to detect QTLs across the mouse genome [7]. Since then additional sets of consomics have been developed: a second set was created from crossing C57BL/6J to the inbred strain MSM/Ms, derived from a Japanese wild mouse, *Mus musculus molossinus* [8]; a third set was developed from crossing C57BL/6J to PWD/Ph [9], an inbred strain established from a pair of trapped wild mice of Mus musculus musculus origin [10].

## 4 Recombinant inbreds and crosses between inbred strains: intercrosses and backcrosses

Mapping to a chromosome is a start, but a long way from identifying a gene. I introduced the method first in this discussion as a heuristic for mapping in general, and while it is a starting point now for some investigators [11], it is still an uncommon approach. Mapping within a chromosome relies on recombination; again inbred strains play a critical role in the production and analysis of recombinants.

**Fig. 3:**
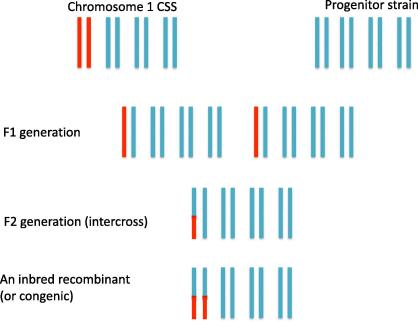
The results of crossing a chromosome 1 consomic to its progenitor. Only four chromosomes are shown. The chromosomal structure is shown by colour, so that blue indicates descent from 129S1/SvlmJ and red from C3HeB/FeJ. Production of the inbred recombinant shown at the bottom of the figure is assumed to take a number of generations (i.e., it is not simply the product of intercrossing the F2 shown in the figure).

Suppose we cross the chromosome one substitution strain to 129S1/SVlmJ and then mate the offspring to each other (an intercross) (Fig. 3). Recombination between the identical chromosomes will occur, but has no effect on the structure of the offspring’s chromosomes. However, recombination on chromosome 1 produces a chromosome with a patchwork of 129S1/SVlmJ and C3HeB/FeJ sequences. If we had animals with the same recombinant chromosome 1, for example in the middle of the chromosome so that lower half is all 129S1/SVlmJ, then we could use the phenotype comparison method described above. We compare them to the CSS with the intact C3HeB/FeJ chromosome and if we find a difference in the RBC count, then we know the genetic effect has to lie in the region of sequence difference, i.e., in the upper half of the chromosome (Fig. 3). If there is no phenotypic difference, then the QTL lies in the lower half of the chromosome. Further mapping could proceed by finding animals with recombinants in the top or bottom half of the chromosome, isolating those regions in inbred strains, and testing for a phenotypic difference with the progenitor strain (this process is identical to the creation and testing of congenic strains, described below).

**Fig. 4:**
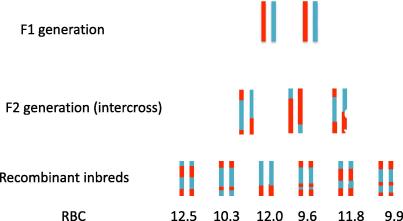
The chromosomal structure of an F1, F2 and of recombinant inbreds. The chromosomal structure is shown by colour, so that blue indicates descent from 129S1/SvlmJ and red from C3HeB/FeJ. Below the recombinant inbreds are shown simulated results for their red blood cell counts (RBC).

Making animals with the necessary recombinants for mapping in this way is far too time consuming, and is not carried out in practice, but it provides an introduction to the use of recombinant inbreds. Fig. 4 shows the result of an intercross, this time using just one chromosome as an example. Siblings are then inbred, which takes about 20 generations, so that they have the chromosome structure shown at the bottom of the figure. Six new inbred strains have been created, each a unique mixture of the two progenitor chromosomes. The chromosome is broken up, but how do we map? Comparison with the progenitor chromosome will not indicate which region contains the QTL since each recombinant chromosome is a patchwork. A significant difference would not tell us which part of the chromosome contains the locus. Instead we take a different approach. We compare the phenotypes of all recombinant chromosomes and find chromosome regions that the high scoring and low scoring chromosomes have in common.

**Fig. 5:**
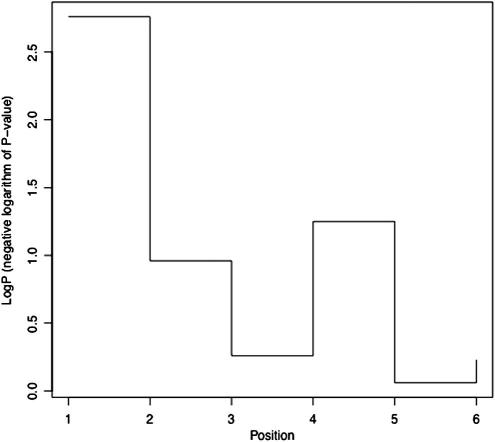
Results of mapping the using recombinant inbreds shown in Fig. 4 at six markers (data are given in Table 1). The *x* axis gives the position in arbitrary units, the vertical axis is the significance of the association, shown as a negative logarithm (base 10) of the *P*-value (log *P*).

At the bottom of the figure is the mean red blood cell score for each strain. The first, third and fifth strains have higher scores than the others; these strains also share blue chromosomal material (from strain 129S1/SvImJ), suggesting that the top of the chromosome contains the locus increasing the phenotype. Of course this might be a chance finding, so we should test the result. We can do this as follows. Assuming we know the origin of each part of the chromosome, as shown in the figure, as either blue (B) or red (R), then we can genotype the chromosome. For example the top most portion of the chromosome the genotype of the first strain is BB, the second RR, the third BB and so forth. Table 1 shows the genotypes for the six strains, along the chromosome, together with the RBC results for each strain. The association between RBC phenotype and genotype can be tested at each location by linear regression. *P*-values are given in Table 1, along with the negative logarithm of the *P*-value (base 10), an easier measure to use for many applications, including plotting the results as shown in Fig. 5. The log *P* is highest (*P*-value therefore lowest) at the left of the figure, and this is where the locus is most likely to be (but note the positions given in the figure correspond to the different genotypes rather than to an absolute position on the chromosome).

The principle illustrated by this simple example can be applied to all chromosomes, not just the one shown in Fig. 5, and provides a method to map genes to sub-chromosomal segments. The basic approach, of obtaining genotypes along the chromosome and testing their associating with the phenotype, applies to the other commonly used methods – mapping using intercrosses and backcrosses.

Fig. 4 shows mice produced from an intercross. Like the recombinant inbreds, they have chromosomes derived from the two inbred progenitors and like the recombinant inbreds they can be genotypes along the chromosome. However, unlike the recombinant inbreds they have heterozygote genotypes (RB), so that a QTL with any mode of action can be detected (dominant, recessive or additive). Also unlike recombinant inbreds the animal’s genotype cannot be reproduced, so there is a limit to the number of phenotypes that can be tested on the same genotype. Backcrosses lack homozygote genotypes of one progenitor, and have fewer recombinants (the recombination density is half that of an intercross), features that simplify the genetic architecture.

## 5 QTL location

The example illustrates a number of problems that will recur throughout this article, some of which have been solved, some not. The first problem is deciding the most likely position of the QTL. Suppose we have results from two markers on the chromosome, both of which are significant. Is the locus in the middle of the two markers, or closer to one or the other, or possibly outside the interval? This problem has also been solved, using methods that probabilistically reconstruct genotypes in intervals between markers, assuming that the location of crossovers is random (that there is no cross-over interference). The first solutions used maximum likelihood and estimate support for a QTL at a particular recombination fraction (denoted by *c*) from the marker compared to a model assuming no QTL [12]. Since this method gives two likelihoods, a natural way of representing the result is a likelihood of odds or LOD score. The LOD is the logarithm (base 10) of the likelihood ratio test statistic for a particular value of the recombination fraction (*c*):

LOD=LR(c)/2ln10=∼LR(c)/4.61

Maximum likelihood estimates are normally distributed for large sample sizes so the confidence intervals of the QTL position and the effect size of the QTL can be obtained from the sampling variance of the ML estimates (this is the justification for the one LOD support interval [12] that indicates the 95% confidence interval for the QTL).

Linear regression can be also be used (and is much easier to implement) [13,14]. Assuming the phenotype is normally distributed, the regression estimates are equivalent to the maximum likelihood estimates. Again a test statistic is calculated at different recombination fractions from a marker and the location that explains most of the variation in the phenotype (where *r*^2^ is maximal) is taken as the most likely position of the QTL.

More recently algorithms for hidden Markov models (HMMs) have been developed [15], which have the advantage that they can take into account genotype errors and partially informative markers. Markov chains are sets of random variables whose states are conditionally independent. A set of observed variables (the observed genotypes) is assumed to depend on a set of unobserved, hidden variables (the true underlying genotypes). An initial probability given by the segregation model defines the initial hidden state; transition probabilities give rise to the distribution of the hidden Markov chain and are a function of recombination fractions; the observed genotypes are then given by a set of emission probabilities (that can include genotyping error and any other factors thought to be important). In QTL mapping the QTL genotypes are simulated via their joint distribution conditional on the observed data probabilities. Further descriptions of maximum likelihood, regression and HMM methods are available in Lynch and Walsh [16] and Broman and Sen [17].

## 6 Significance thresholds

The second problem concerns the significance of the result. The *P*-value in the example in the table (*P* < 0.002, log *P* of 2.8) exceeds the accepted threshold of *P* < 0.05, but does not take into account the number of tests carried out. Six tests were performed. With no significant difference between genotypes each test has a 1 in 20 chance of yielding a result less than 0.05, so if we carried out 20 tests we would expect at least one to be less than 0.05. Dividing the *P*-value by the number of tests carried out is a simple solution (this is a Bonferroni correction): the threshold then becomes 0.05/6 = 0.008, which is exceeded in our example. Screening the genome would mean testing more regions, so the significance threshold will be much smaller, but the same principle applies.

There is a complication to this simple approach. Typically, we do not know the position of recombination break points; they are inferred from the genotypes of markers to distinguish the strain origins of the chromosome. Since in an F2 or a backcross each chromosome undergoes at most a handful of recombinants in the single generation that scrambles the progenitor lines, a small set of markers is sufficient to screen each chromosome (a few hundred markers can be used to screen the genome). However, markers may often detect the same recombinants, so that the information we obtain is, partly redundant. Put another way the genotypes are correlated, due to linkage disequilibrium, so that the statistical tests we carry out in mapping are not independent, and the Bonferroni correction is too strict.

The problem of the dependency structure of statistical tests, and an associated problem that the tests often assume a specific phenotypic distribution (normality), can be dealt with by permutation. Churchill and Doerge proposed repeated shuffling of the trait values at random amongst the test subjects animals and re-analysis of each shuffled data set [18]. This procedure generates a set of *P*-values, whose distribution corresponds to that expected if there were no detectable QTLs in the data. For example to obtain the significance threshold for the data in Table 1, I re-order the phenotypes in relation to the genotypes and re-analyse the data, obtaining a new set of *P*-values (0.54, 0.27, 0.35, 0.64, 0.19 and 0.82). I keep the most significant result (0.19) and I repeat this process thousands of times, each time keeping the most significant result. I then ask how often I see, amongst the thousands of *P*-values I have generated, results that are equal to, or more significant than, the most significant *P*-value found in my data without re-arrangement. I find that about one in every of 100 of the permuted results are more significant than 0.002, so my empirically derived *P*-value is about 1%.

While this method is robust and easy to implement, it makes one critical assumption: that the animals are equally genetically related [19]. It will not work for example when the data consist of a set of families. Permutation among siblings will work (as they are all similarly related) but not between families. Methods for dealing with structured populations are available, and described later; here the point to emphasize is that permutation cannot be applied to all experimental designs (for example it is not appropriate for advanced intercross lines or heterogeneous stocks).

## 7 Multiple QTLs

The mapping methods described above assume a single QTL is associated with the phenotype. In fact the majority of quantitative phenotypes arise from the joint effect of multiple loci. The models described above test for the presence of a single locus, one interval at a time, without taking into account the presence of other QTLs (their effect is mopped up in a residual variance term). Why is it bad to look for one QTL at a time?

The problem is that ignoring the conjoint effects of multiple QTLs can bias the results. This happens in two ways. First linked QTL, for example two or more QTLs in the same, or adjacent marker intervals, can produce spurious peaks. Sometimes what appears to be the largest peak is just an artefact of the mapping. Second, unlinked QTL contribute to the phenotypic variation, in effect increasing noise and reducing the power to detect loci.

Dealing with multiple QTLs is the third problem, after location and significance thresholds, and it has been less well solved than the other two. Composite interval mapping [20,21] and multiple interval mapping [22] were introduced as ways to model multiple QTLs, but they face the difficulty of having to cover an enormous number of models: assuming just 100 markers and a simple additive model there are 3^100^ models in an intercross (three because there are three genotypes) and exponentially more possibilities when we allow for interactions. Forward and backward selection can be used to reduce the search space (by adding in markers one at a time and discarding those that do not increase the explanatory power of the model), but results can vary depending on the order in which markers are added.

An alternative solution is to use Bayesian QTL mapping in which a prior distribution of QTL models, QTL positions and effects is set and a posterior estimated, given the data (see for example [23,24]). Despite their forbidding complexity, and the unusual terminology in which they are cloaked (posteriors and priors for example) Bayesian approaches are much closer to how we weigh up evidence for and against a hypothesis. Rather than estimating the probability that a null hypothesis is incorrect, they start with reasonable estimates of the parameters (number of loci and effect size; these are the prior probabilities) and provide estimates that these are correct (these are the posterior probabilities). Estimating the posterior distribution is too complex to solve analytically and so is usually approximated by sampling from a Markov chain whose limiting distribution is the target posterior distribution (a Markov chain Monte Carlo (MCMC)). Bayesian approaches avoid the difficulty of testing an unknown number of QTLs with unknown effect sizes and unknown interactions: all these can be estimated from the posterior probability. However, specifying the prior distributions is hard, as is choosing the appropriate MCMC algorithm, the computational demands are intense (I have spent 3 weeks running a model for three loci among 100 markers), and the interpretation of the posterior is not straightforward. But as the methods are sill in their infancy it is likely that they may yet displace the classical frequentist approaches described above.

## 8 Mapping resolution and the problem of gene identification

**Fig. 6:**
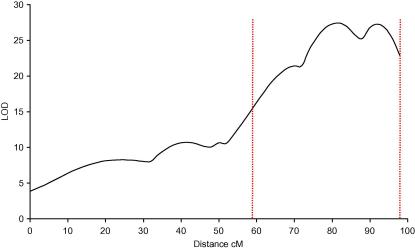
Mapping QTLs using an intercross: results on a single chromosome. The vertical axis shows the significance of the association expressed as a likelihood of odds or LOD score. In an intercross the LOD score is identical to a log *P* (negative logarithm (base 10) of the *P*-value) so the peak can be read as having a *P*-value of 10^−25^. The dotted red lines indicate the extent of the 95% confidence interval.

All of the three problems described above, QTL location, significance threshold and modeling multiple QTLs, have an impact on our ability to find genes. They will recur throughout the subsequent discussion. Consider first Fig. 6, which shows the results of mapping a QTL onto a single chromosome using an intercross design. The peak of the locus is at 85 centimorgans but the 95% confidence interval is very broad – in fact encompassing about 40 centimorgans. Given an average gene density of 10 genes per megabase and that in the mouse one centimorgan is about 2 megabases in size, about 800 genes are potential candidates.

Initial approaches to gene identification relied upon the construction of congenics as a way to reduce the interval. A congenic is an inbred strain that contains one segment of a chromosome from one strain, all the rest from the other, as shown in Fig. 3. Repeated back crossing to isolate increasingly smaller segments could, in theory, allow the identification of the gene involved, an idea that still motivates attempts at QTL cloning. And while there have been some successes using this approach [25], a much commoner outcome is the discovery that there is no single effect, but multiple, physically linked small effects. There are a large number of examples of this phenomenon, including QTLs influencing seizures [26], obesity [27], growth [28], blood pressure [29–33], diabetes [34], antibody production [35] and infection [36].

Alternative approaches to gene identification have taken three routes. The first of these is to use recombination to break up chromosomes so that there is less linkage disequilibrium (LD) (in other words lower correlation between genotypes at different markers). Simply put, lower LD means higher resolution, which comes at the price of needing more markers to screen the genome, but this objection is balanced, if not outweighed, by falling genotype costs. Two sources of additional recombinants have been used: more animals and more generations.

## 9 The Collaborative Cross

Until recently the number of recombinant inbreds (RIs) available to mouse geneticists has been relatively small. The widely used panel of BXD RIs consists of 26 lines. QTLs have to account for more than half the additive genetic variance to be detected in the BXD and are mapped into regions of tens of megabases. Increasing the number of RIs increases the power to detect a QTL and also increases resolution [37]: a panel known as the LXS (because derived from long and short alcohol induced sleep) consists of 77 strains is estimated to map QTLs into regions with 2–20 Mb resolution [38] and the development of a novel set of 46 BXD lines is estimated to have doubled the number of recombinants [39].

In addition to these panels, other inbred resources have been created for mapping. Subconsomic strains have been created by transferring segments of individual autosomes of DBA/2J onto C57BL/6J [40] (95% of autosomes are covered); 40 subconsomic strains were created from CAST/Ei crossed onto C57BL/6J [41] (80% of CAST/Ei autosomes are covered). An additional resource comes from the creation of interspecific recombinant congenic strains between C57BL/6 and mice of the Mus spretus species [42].

However, the most ambitious attempt to provide a high-resolution mapping resource is the development of the Collaborative Cross (CC) [43]. The CC is descended from eight divergent strains of mice: five classical inbred strains (A/J, C57BL/6J, 129S1/SvImJ, NOD/LtJ and NZO/H1LtJ) and three wild-derived strains (CAST/EiJ, PWK/PhJ and WSB/EiJ) were selected as founders of the CC. In each mouse the average distance between recombinants is approximately 12 Mb, and the QTL mapping resolution is expected to be about a megabase [44].

The CC has generated considerable interest not only because it is expected to be the largest RI panel created, with more than 1000 lines being inbred [45–47]. It has another unique feature: it was designed to capture more genetic diversity than other resources. This is important because a resource that has variation distributed throughout the genome can be used to interrogate the effects of all genes on a phenotype. It avoids having blind spots, regions of the genome where there is no variation. A systematic breeding design used to create the CC ensures the genetic contribution of each of the eight founder strains is equivalent, so that, when averaged across all CC lines, the allele frequency at each locus will be approximately 0.125. Analysis of the CC indicates that its distribution of polymorphism is representative of natural populations and that it is suited to systems biology applications analysis [48].

Once genotyped, mapping of almost any conceivable trait will be possible in the CC. And since the same genotype will be interrogated, an almost limitless amount of information can be accumulated, from molecular through to the level of the whole organism. Furthermore, F1 hybrids created from pairs of CC lines will be a reproducible source of heterozygous animals, therefore modeling what is found in wild populations. The possibility of tracing the impact of genetic variation, through cellular and organ level variation to whole body phenotypes is exciting; realizing this potential will require large-scale phenotyping, on the scale currently employed of analysing the products of mutagenesis experiments [49] and the genome-wide knockouts. Maintaining the 1000 or so CC lines poses significant challenges.

Logistic issues aside, there are two scientific issues that remain unresolved. The first is how data from the CC can be used to identify genes. Mapping to the resolution of a megabase will identify about 10 genes, and though the use of F1s can be used to reduce the interval, it is unlikely that creating crosses will be a practical solution given the extremely large number of loci that will need to be dissected. The second is how best to integrate the multiple sets of data to address biological questions. An example of how this might be done is found in work integrating expression and phenotypic data, where attempts have been made to model causal relationships between genotype, transcript abundance and phenotypic variation [50,51]. This point is discussed later.

## 10 Historical recombinants: advanced intercross lines

**Fig. 7:**
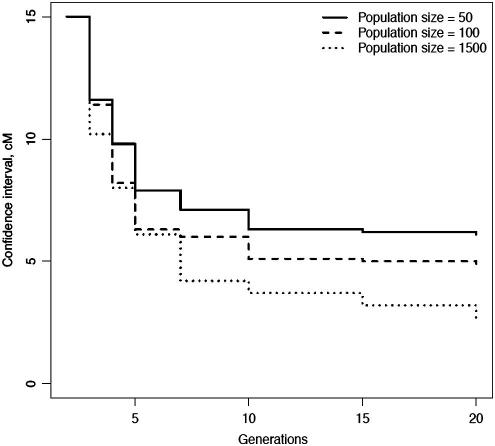
Simulation results for the confidence interval of a QTL, showing the effect of increasing the number of generations (*x* axis). The confidence interval is plotted on the y axis in centimorgans (cM). The effect of changing population size is also shown, with results for three breeding populations of size 50, 150 and 1500.

Continued intercrossing reduces linkage disequilibrium and, with increasing numbers of generations the proportion of recombinants between any linked loci approaches 0.5, in other words approaches linkage equilibrium [52]. Thus one way to increase mapping resolution is to use a cross that has accumulated recombinations over many generations [53,54]. For instance, rather than mapping in an F2, map in an F10. Darvasi and Soller showed that so doing would increase mapping resolution fivefold; there are diminishing returns from using later generations. As shown by simulation (Fig. 7), this depends in part on the population size, but given the time needed to create even an F10 laboratories have not used advanced intercross lines (AILs) with more generations.

AILs have been used to fine-map collagen-induced arthritis QTLs [55], fear-related behaviour [56], lung cancer susceptibility [57], high density lipoproteins [58] and trypanosomiasis resistance [59]. The method certainly reduces the size of the QTL confidence interval but suffers from two problems. The first is that genetically relatedness between individuals in a population of AILs varies, unlike an F2 where all animals are siblings. There is population structure that violates assumptions about independence upon which some genetic analyses depend. This is particularly bad when, as often happens, AIL populations used for mapping are generated from multiple litters from the same families. False positives will occur if structure is ignored: long-range correlations between genetic markers occur so that it is sometimes possible to predict the genotype of a marker on one chromosome by the genotype on another. These long-range correlations are due to partial fixation of pairs of haplotype blocks within subsets of the population. There are solutions to this problem, correcting for genetic relatedness using mixed model association [60] or model averaging approaches [61], although their implementation can be daunting. The second problem is more intractable: resolution is still insufficient to identify genes. To date, no AIL experiment has led to the identification of a gene at a mouse QTL.

## 11 Historical recombinants: heterogeneous stocks

Heterogeneous stocks (HS) are an extension of AILs, in that they are outbred mice derived from inbred strains, but there are two differences: HS descend from more than two strains (eight in the extant stocks) and the number of generations of breeding is many more than for AILs (greater than 50). The latter means that HS have higher mapping resolution: QTLs typically are mapped into intervals of less than three megabases [62]. An HS can deliver higher mapping resolution than an AIL because of the larger number of differing chromosomes with which the stock is seeded, but, as with the AIL, there are diminishing benefits with increasing generations. To some extent this can be alleviated by a larger population size, since, assuming random mating, the time required for a neutral allele to go to fixation in a population is approximately equal to four times the effective population size [63].

In practice HS are maintained with no more than 40 mating pairs. These small breeding populations used to maintain an HS mean that, as with the AIL, analyses of an HS have to take into account population structure, and the same solutions apply [60,61]. Note that model averaging mapping results are not returned as a *P*-value of the association between phenotype and locus. Rather we obtain the expected proportion of times a genetic predictor is included in a multilocus model, given by a Monte Carlo estimate, termed a resample model inclusion probability (RMIP). An RMIP of 1 would indicate that the locus is included in every resampling, an RMIP of 0.5 that the locus is included in half the resamples and so on. This does not easily translate into a false positive or false negative value and unfortunately the meaning of an RMIP is not universal: it depends on the experiment. Simulation is necessary in order to interpret it as a probability of a QTL and different mapping populations require individual calibration.

There are currently two mouse HS available. The oldest was first bred by McClearn and is an eight-way cross of C57BL/6, BALB/c, RIII, AKR, DBA/2, I, A/J and C3H inbred mouse strains [64]. The more recent, established by Hitzemann, descends from A/J, AKR/J, BALBc/J, CBA/J, C3H/HeJ, C57BL/6J, DBA/2J and LP/J [65]. The strain origins are important since they are used in mapping and for this reason the Hitzemann HS is preferred since the strain designations of the McClearn stock are not unambiguous. DNA from all progenitors is needed for mapping in an HS. Markers used for genotyping are usually single nucleotide polymorphisms (SNP) and so have fewer alleles (two) than the number of haplotypes segregating in the cross (eight). Consequently genotyped markers do not unambiguously identify the underlying strain haplotype with the result that observed genotypes may not reflect the underlying haplotypes. This means that QTLs can be missed by single marker association analysis that ignores strain haplotypes [66]. A solution to this problem is reconstruct haplotypes, based on the recombination distance between markers and the known progenitor alleles. For this to work a good recombination map is needed [67] and access to the genotypes of the progenitors. Mapping then occurs by associating the probabilistically reconstructed haplotypes at each locus in the genome with the phenotype [66]. Evidence for the location of QTLs is then provided within a framework that takes into account population structure. Small effect QTLs have now been mapped to subcentimorgan resolution using heterogeneous stock mice [62,66,68–70].

## 12 Historical recombinants: outbreds

None of the approaches described before gives gene-level resolution. In some species, in outbred populations linkage disequilibrium is often low enough to map genetic effects into regions of a few kilobases. There have been few analyses of the genetics of wild mice [71], but an assessment of linkage disequilibrium in mice caught in Arizona demonstrated that the decay in linkage disequilibrium with physical distance is comparable to that found in human populations: the 95th percentile of the genotype correlation (*r*^2^) decreases to less than 0.4 within 100 kb [72].

The LD structure of wild mice indicates that it would be possible to map to the level of individual genes, using mapping strategies developed in human genome-wide association studies. Conceptually, the methodology is an extension of the methods previously described, except now employed on a grand scale. Each marker is tested for association with the phenotype, for example by analysis of variance, but because of the low LD rather than needing a few hundred markers (as in an F2 intercross) or a few tens of thousands of markers (as in an HS analysis), a genome-wide analysis needs millions of markers to obtain reasonable coverage. Complications arise due to variation in LD and variation in allele frequency, but many of analytical the issues have been resolved and appropriate software developed (see for example [73]).

The use of wild mice for mapping quantitative traits is likely to suffer from all of the same problems that afflict association mapping in human studies [74]: tens of thousands of subjects will be needed for robust detection of common causal variants and the majority of the genetic variance is likely to remain unexplained, even using large sample sizes, because wild populations are likely to contain large numbers of rare variants contributing to phenotypic variation. And wild mice present problems of their own: phenotyping will be difficult (their health status forbids their import into any clean animal facility), behavioural testing will be particularly challenging; there is also evidence for population structure [72].

One alternative is to use outbred mice from commercial suppliers such as Harlan and Charles River Laboratories. The colony sizes are often in the thousands (animals are used by pharmaceutical companies for toxicology experiments and often need thousands of animals), so that there is less likelihood of the complications that affect AIL and HS populations. LD in some outbred stocks (MF1) has been shown to allow high-resolution mapping [75]. Outbred CD1 mice have been used to fine-map a susceptibility locus for pulmonary adenoma [76]; outbred MF1 were used to identify *rgs2* as a gene influencing anxiety [77].

However, mapping resolution is not the only measure of the likely usefulness of the mice for mapping genes. Low LD occurs in large populations maintained for many generations but an unfortunate corollary is the presence of rare alleles. This is because allele frequencies drift to extremes and new, rare, alleles arise as a consequence of mutations. The more rare alleles in a population, and the more they contribute to phenotypic variation, the more difficult it will be to detect QTLs using genome-wide association strategies that genotype only common alleles.

Most outbred stocks are known to derive from animals from a single population, such as the ‘Swiss’ stocks which descend from two male and seven female imported from Lausanne, Switzerland [78]. Available sequencing data indicates that commercially available outbred stocks originated from mice genetically similar to inbred strains and that almost all of the genetic variants can be found in classical laboratory strains [77]. Haplotype reconstruction demonstrates how variants in the outbreds can be modeled as descending from inbred progenitors [77]. The limited sequence diversity means it is possible to impute the sequence of any commercially available mouse from the sequences of inbred strains. Thus the full catalogue of sequence variation in a stock could be obtained by sequencing the inbred strains presumed to be founders for it, and genotyping the stock at a skeleton of SNPs. Therefore, we should be able to detect the effect of all variants, a situation that has so far eluded studies in completely outbred populations.

## 13 Historical recombinants: inbreds

Another source of recombinants lies in the origin of laboratory strains. Just as AIL and HS are descended from a small number of chromosomes, the inbred strains themselves are the product of a small number of progenitors [79,80]. The common laboratory strains have a complicated origin that has left an imprint on the structure of their chromosomes.

Distinct subspecies of *M. musculus* are present in largely non-overlapping regions of the world [81]. Wild house mice in Asia (*M. musculus musculus*) and in Europe (*M. musculus domesticus*, also present in the Americas and Australia following human emigration) diverged about 1 million years ago. Apart from a hybrid zone in Eastern Europe, the two subspecies live separately. Mice from Asia (*musculus*, *castaneus* and *molossinus*) were bred with European (*domesticus*) subspecies to produce fancy mice in the nineteenth century, and inbred in the twentieth inbred to create laboratory strains of mice. Consequently, laboratory mice have genomic segments derived from different subspecies of *M. musculus* and from different chromosomes in these subspecies.

Since a QTL must be contained in a region where sequence divergence corresponds to genetic action, QTLs can be excluded wherever there is identity by descent, and included in regions where the strain distribution pattern of sequence variants agrees with that of the QTL alleles [68,82–84]. It should be possible to map QTLs by treating laboratory strains as a set of recombinant inbreds, whose chromosomes have a very fine-grained mosaic structure, potentially providing very high level mapping resolution [85,86].

The pattern of sequence variation between inbred strains initially suggested that the distribution of polymorphisms had a relatively simple mosaic structure, consistent with the derivation from a small number of Asian and European stocks [87–89]. We now know that the picture is more complex [90–92]. Using a set of 8.3 million SNPs two groups performed a detailed reconstruction of the phylogenetic reconstruction of laboratory mice [93,94]. Comparisons between classical strains found that between 36% and 62% of the genome was identical by descent. Among the 11 classical strains analysed, about 10% of the genome was found to be identical by descent. On average, 9% of the genome had a different subspecific origin between any given pair of classical strains, whereas 91% of the genome shared the same subspecific origin.

In principle, mapping using the inbred strains is just like mapping using recombinant inbreds: by establishing phylogenetic relationships amongst inbred strains from sequence information, it is possible to identify regions that are identical by descent. The problem is that in contrast to a set of recombinant inbred strains where genotyping unambiguously defines descent so that genetic identities can be associated with phenotypic similarities, finding sequence variants shared by a set of inbreds does not guarantee their descent from a common ancestor. Results from the SNP studies described above indicate that the most common strain distribution patterns in classical strains are found on average in almost half of mouse chromosomes, which will give rise to false positives in genetic mapping studies [93,94].

The low power of using inbreds in this way (sometimes called *in silico* mapping) has been shown by a number of groups [95–97]. As with AIL and HS, the complex relatedness requires methods that deal with population structure [60,61]. There are, unfortunately, a number of studies, that have not applied such methods, so that it is not clear how many of the reported successes of mapping are false positives [98,99].

The statistical power of *in silico* mapping is only high for phenotypes when the background genetic effect is low or for large genetic effects [97]. There is little doubt that *in silico* mapping is very effective for monogenic highly penetrant mutations [85,87,100]. The problem is that *in silico* mapping does not perform well in detecting small effect loci [100]. As discussed later, almost all QTLs are small effect. Current attempts to increase the power of *in silico* approaches involve increasing sample sizes (combining 29 classic inbred strains and three sets of recombinant inbred (RI) strains to obtain 100 lines [101]) and imputing high density SNPs on 94 strains [97]. These improvements have indeed detected smaller effects, but the strength of the signal remains weak.

## 14 Genes: knock outs, complementation and transcripts

Genetic mapping, even if carried to the resolution of a single nucleotide, does not identify genes, only functional variants, which may be at some distance, physically, from the genes they influence. Where the variant interrupts a protein-coding gene, or otherwise disrupts gene function in a recognized fashion, knowing the sequence variant will aid gene identification (though it does not prove it). One of the clear results from high-resolution mapping in humans is that QTLs do not lie in coding regions [102], often in fact they lie completely outside genes, in regions whose function remains obscure. There is every reason to suppose that the same will be true of the molecular basis of quantitative traits in mice. This makes gene identification hard.

One solution is to work at the level of genes, rather than sequence variants. A simple test that a gene is involved in a phenotype is whether a knockout has an abnormal phenotype [103]. The availability of large numbers of engineered mutations makes this approach relatively easy, though time consuming (especially if the knockout is only available as an embryonic stem cell line). But it does not prove that the QTL acts either at or through the gene. QTL alleles as mentioned above are rarely knockouts; they alter gene function in other ways, which are not necessarily modeled by the null allele, and there are plenty examples in genetics where contrasting phenotypes can be attributed to different alleles at the same gene [104]. Moreover some phenotypes, such as height or weight, are influenced by so many gene that upwards of a third of all knockouts may show a phenotype [105]. Relying on knockouts to implicate a gene at a QTL can therefore give both false negative and false positive results.

However, knockouts can be used in quantitative complementation tests, or QTL-knockout interaction [106] to test the candidacy of a gene at a QTL. The test is not complementation in its classical form because it is testing for an interaction between the null allele and the QTL, rather than for a main effect of either and for this reason the name QTL-knockout interaction test is preferable [107]. An inbred animal bearing one QTL allele (for example ‘high’) is mated to an inbred animal with a null allele of the gene of interest (‘*m*’) and also to the co-isogenic wild type animal (‘*wt*’). A similar pair of crosses is established, but this time using an inbred strain with the alternative QTL allele (‘low’). If the difference in mean phenotype between the high/*m* and low/*m* genotypes is greater than that between the high/*wt* and low/*wt* genotypes there is evidence of quantitative failure of the mutation to complement the QTL alleles. This is detected as a statistical ‘Cross’ (*m* or *wt*) by ‘Line’ (high or low) interaction in a two-way analysis of variance.

A significant interaction indicates that the *wt* gene is under the control of a QTL allele on the homologous chromosome. It should be noted that the test does not implicate any particular QTL, which could be anywhere on the genome where the high and low strains differ. The method has been used to identify one gene (*Rgs2*) [77] but has yet to be applied in a systematic fashion to the thousands of QTLs discovered in the mouse.

An alternative that has gained considerable attention is to look for an association between variation in phenotype and transcript abundance. A significant association implicates the gene responsible for the transcript, but it is possible that the phenotype alters transcript abundance, rather than the other way round, so on its own the association does prove that the gene is casually related to the phenotype. Comparing a statistical model in which genetic variation contributes to transcript variation and that in turn contributes to phenotypic variation, with alternative non-causal models, is one way to implicate a gene [51]. This approach can be extended by associating an entire network of transcripts to a phenotype [50] thereby moving beyond just finding a gene to identifying a mechanistic pathway. For example variation in a macrophage-enriched metabolic network of transcripts with correlated expression was found to have a causal relationship with obesity, diabetes and atherosclerosis-associated traits [50].

Schadt argues that the identification of the macrophage-enriched metabolic network highlights several important features of the network approach to understanding disease: “first, the network analyses revealed hundreds of disease-causing genes acting together in coherent networks; second, within a given network supported as being causal for disease, perturbing individual genes supported as being causal for disease affected the state of the network; and, third, DNA and other sources of variation in one species can be used to construct disease networks that are relevant in a second species and that act as sensors for many sources of variation” [108]. The emphasis on large-scale networks as functional units pushes gene identification to one side, moving closer to biological mechanism. But to do so requires interrogating expression of the entire genome, presumably in multiple tissues at multiple time points, with correspondingly huge data sets requiring sophisticated mathematical algorithms for their interpretation.

Apart from the forbidding analytic complexity of this approach (we lack measures of the sensitivity and specificity of the causal modeling method) it remains unclear to what extent linkage disequilibrium between QTL and variants that alter expression in neighbouring genes could account for the results: at least part of a network of correlated gene expression could arise because of LD between SNPs in the promoters of genes of unrelated function that happen to lie next to each other on the chromosome.

## 15 Genetic architecture of quantitative traits in mice

Genetic mapping has identified thousands of QTLs in mice; gene identification remains slow, and in very few instances do we know the nature of the sequence variation that gives rise to quantitative variation in the mouse. However, we do now know a considerable amount about the genetic architecture of complex traits. This information is important, not merely in its own right, but because it should help design studies that identify genes. In this section, I summarize briefly what we know. More details are given in [102].

**Fig. 8:**
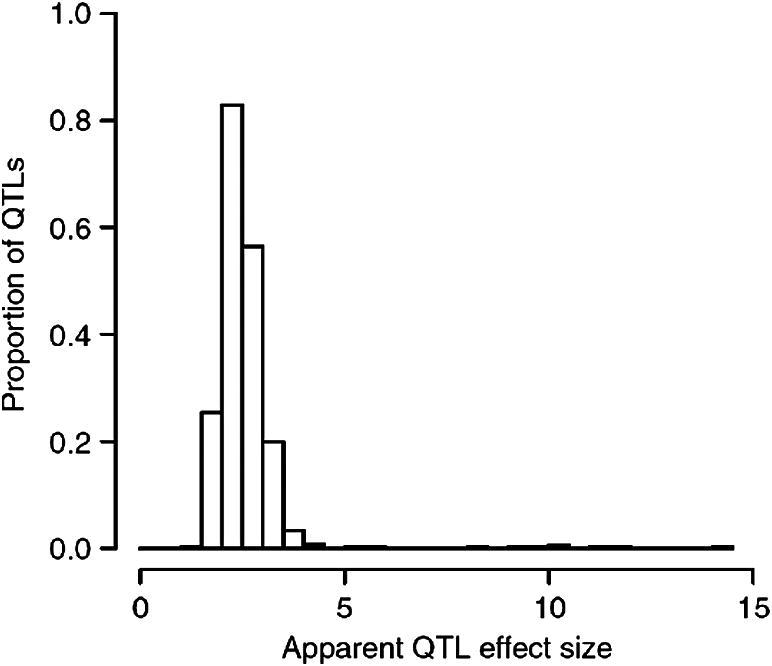
The effect size (expressed as the proportion of variance attributable to the locus) for 843 QTLs mapped in an HS population of mice. Data are from [62].

First the effect sizes of QTLs are small. Fig. 8 plots the effect sizes of about 900 QTLs detected in an HS experiment [62]. The phenotypes include a range of physiological, anatomical, haematological, biochemical and immunological traits. With very few exceptions the majority of QTLs explain less than 3% of the variation in a phenotype. This is larger than the average effects found in completely outbred populations, because of the reduced genetic diversity in the HS mice, but less than in inbred strain crosses where a similar summary of many phenotypes finds an average of QTL effect size of about 5% [109].

**Fig. 9:**
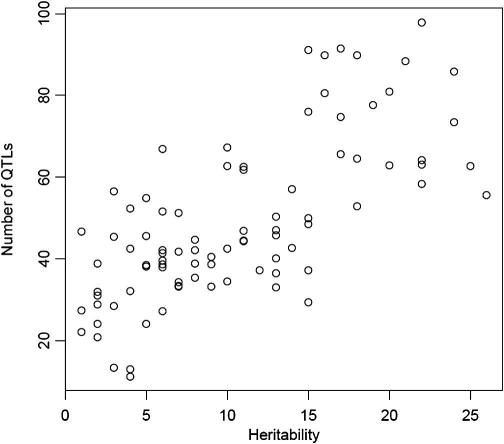
The relationship between heritability, plotted on the *x* axis, and the number of QTLs detected, plotted on the *y* axis. Data are from 843 QTLs mapped in an HS population of mice [62].

Fig. 9 compares the heritability of each phenotype (on the horizontal axis) to the number of QTLs detected [62]. There is a clear linear relationship: the larger the heritability the more QTLs detected. This means that mapping a highly heritable phenotype increases the chances of detecting a QTL because there are more loci to detect, rather than that large effects are more prevalent.

**Fig. 10:**
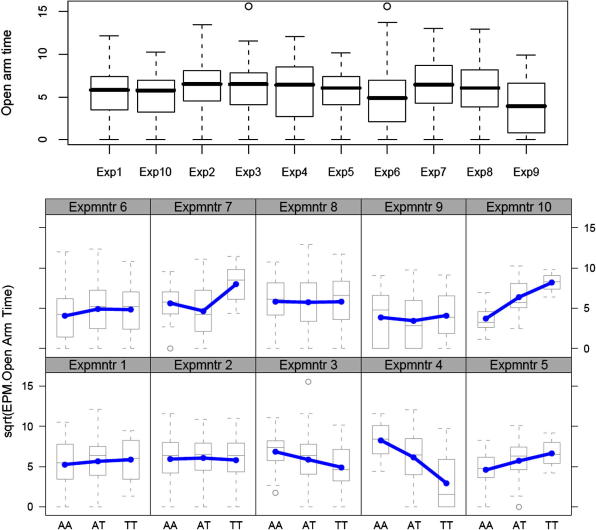
Context dependent genetic effects. The top panel shows the effect of experimenter (plotted on the *x* axis) on a fear-related measure in mice, time in the open arms of an elevated plus maze, plotted on the *y* axis. The bottom panel shows the effect is dependent on genotype. Results are shown for a single marker with three genotypes (AA, AT and TT) shown on the *x* axis. The distribution of the phenotype associated with each genotype is shown separately for each experimenter.

Second, context dependent effects are very common [110]. The top panel of Fig. 10 shows the effect of experimenter on a fear-related measure (entries into the open arms of an elevated plus maze). There is variation between experimenters and overall the effect is significant but the effect is relatively small, accounting for about 5% of the variation. However, the effect depends in part on the genotype of the animal. This is illustrated in the bottom panel of Fig. 10 where it can be seen that experimenter 10 elicits a large genetic effect; experimenter 2 none at all. Also the direction of effect differs: at the hands of experimenter 10 and 5 animals with the TT genotype become more anxious, but become less anxious the hands of experimenter 4. Overall the interaction effect is large, at least twice as large as the effect of experimenter alone. A survey of environmental effects, indexed by variation in the time when a phenotype was measured, showed that the effect of interaction is extremely large: in aggregate the total effect of interactions with the environment is at least as important as that of immediate heritability [62].

The small size of the effects, together with their context dependency, are indications of the challenge facing attempts to identify quantitative trait genes in the mouse. It is unlikely that any single method will be appropriate, no single resource will meet every investigators needs. However, the shift towards interest in systems approaches, coupled with the falling costs of whole genome sequencing, and the development of new complex trait resources, may allow significant inroads to be made into one of the most difficult problems in mouse genetics: understanding the molecular basis of common, quantitative variation.

## Figures and Tables

**Table 1:** The data are from a simulated experiment, where mice are genotyped at a diallelic locus (with alleles R and B) and tested for association with variation in red blood cell count (RBC). The significance of the result is shown both as a *P*-value and as a negative logarithm (base 10) of the *P*-value (log *P*). The markers are given arbitrary names (Pos. 1–Pos. 6) representing their order on the chromosome.

RBC	Pos. 1	Pos. 2	Pos. 3	Pos. 4	Pos. 5	Pos. 6	*P*-value	log *P*
12.5	BB	RR	BB	BB	BB	RR	0.002	2.76
10.3	RR	BB	BB	BB	RR	BB	0.111	0.96
12	BB	BB	BB	BB	RR	RR	0.547	0.26
9.6	RR	BB	BB	RR	BB	RR	0.057	1.25
11.8	BB	RR	RR	BB	BB	RR	0.874	0.06
9.9	RR	BB	BB	RR	BB	RR	0.583	0.23

## References

[1] Paigen K., Eppig J.T. (2000). Mamm. Genome.

[2] Bogue M.A., Grubb S.C., Maddatu T.P., Bult C.J. (2007). Nucleic Acids Res..

[3] Law C.N. (1966). Genetics.

[4] Caligari P.D., Mather K. (1975). Proc. R. Soc. Lond. B: Biol. Sci..

[5] Beamer W.G., Shultz K.L., Churchill G.A., Frankel W.N., Baylink D.J., Rosen C.J., Donahue L.R. (1999). Mamm. Genome.

[6] Nadeau J.H., Singer J.B., Matin A., Lander E.S. (2000). Nat. Genet..

[7] Singer J.B., Hill A.E., Burrage L.C., Olszens K.R., Song J., Justice M., O’Brien W.E., Conti D.V., Witte J.S., Lander E.S. (2004). Science.

[8] Takada T., Mita A., Maeno A., Sakai T., Shitara H., Kikkawa Y., Moriwaki K., Yonekawa H., Shiroishi T. (2008). Genome Res..

[9] Gregorova S., Divina P., Storchova R., Trachtulec Z., Fotopulosova V., Svenson K.L., Donahue L.R., Paigen B., Forejt J. (2008). Genome Res..

[10] Gregorova S., Forejt J. (2000). Folia Biol. (Praha).

[11] Petryshen T.L., Kirby A., Hammer R.P., Purcell S., O’Leary S.B., Singer J.B., Hill A.E., Nadeau J.H., Daly M.J., Sklar P. (2005). Genetics.

[12] Lander E.S., Botstein D. (1989). Genetics.

[13] Knott S.A., Haley C.S. (1992). Genetics.

[14] Martinez O., Curnow R.N. (1992). Theor. Appl. Genet..

[15] Sen S., Churchill G.A. (2001). Genetics.

[16] Lynch M., Walsh B. (1998). Genetics and Analysis of Quantitative Traits.

[17] Broman K.W., Sen S. (2009). A Guide to QTL Mapping with R/qtl.

[18] Churchill G.A., Doerge R.W. (1994). Genetics.

[19] Churchill G.A., Doerge R.W. (2008). Genetics.

[20] Zeng Z.-B. (1994). Genetics.

[21] Jansen R.C., Stam P. (1994). Genetics.

[22] Kao C.H., Zeng Z.B., Teasdale R.D. (1999). Genetics.

[23] Yi N., Banerjee S. (2009). Genetics.

[24] Yi N., Xu S. (2008). Genetics.

[25] Tomida S., Mamiya T., Sakamaki H., Miura M., Aosaki T., Masuda M., Niwa M., Kameyama T., Kobayashi J., Iwaki Y. (2009). Nat. Genet..

[26] Legare M.E., Bartlett F.S., Frankel W.N. (2000). Genome Res..

[27] Stylianou I.M., Christians J.K., Keightley P.D., Bunger L., Clinton M., Bulfield G., Horvat S. (2004). Mamm. Genome.

[28] Christians J.K., Keightley P.D. (2004). Mamm. Genome.

[29] Ariyarajah A., Palijan A., Dutil J., Prithiviraj K., Deng Y., Deng A.Y. (2004). J. Hypertens..

[30] Alemayehu A., Breen L., Krenova D., Printz M.P. (2002). Physiol. Genom..

[31] Garrett M.R., Rapp J.P. (2002). Physiol. Genom..

[32] Garrett M.R., Rapp J.P. (2002). Mamm. Genome.

[33] Frantz S., Clemitson J.R., Bihoreau M.T., Gauguier D., Samani N.J. (2001). Hypertension.

[34] Podolin P.L., Denny P., Armitage N., Lord C.J., Hill N.J., Levy E.R., Peterson L.B., Todd J.A., Wicker L.S., Lyons P.A. (1998). Mamm. Genome.

[35] Puel A., Mevel J.C., Bouthillier Y., Decreusefond C., Fridman W.H., Feingold N., Mouton D. (1998). Immunogenetics.

[36] Bihl F., Brahic M., Bureau J.F. (1999). Genetics.

[37] Belknap J.K., Mitchel S.R., Crabbe J.C. (1996). Behav. Genet..

[38] Williams R.W., Bennett B., Lu L., Gu J., DeFries J.C., Carosone-Link P.J., Rikke B.A., Belknap J.K., Johnson T.E. (2004). Mamm. Genome.

[39] Peirce J.L., Lu L., Gu J., Silver L.M., Williams R.W. (2004). BMC Genet..

[40] Davis R.C., Schadt E.E., Smith D.J., Hsieh E.W., Cervino A.C., van Nas A., Rosales M., Doss S., Meng H., Allayee H. (2005). Genomics.

[41] Davis R.C., Jin A., Rosales M., Yu S., Xia X., Ranola K., Schadt E.E., Lusis A.J. (2007). Genomics.

[42] Burgio G., Szatanik M., Guenet J.L., Arnau M.R., Panthier J.J., Montagutelli X. (2007). Genetics.

[43] Churchill G.A., Airey D.C., Allayee H., Angel J.M., Attie A.D., Beatty J., Beavis W.D., Belknap J.K., Bennett B., Berrettini W. (2004). Nat. Genet..

[44] Valdar W., Flint J., Mott R. (2006). Genetics.

[45] Iraqi F.A., Churchill G., Mott R. (2008). Mamm. Genome.

[46] Morahan G., Balmer L., Monley D. (2008). Mamm. Genome.

[47] Chesler E.J., Miller D.R., Branstetter L.R., Galloway L.D., Jackson B.L., Philip V.M., Voy B.H., Culiat C.T., Threadgill D.W., Williams R.W. (2008). Mamm. Genome.

[48] Roberts A., Pardo-Manuel de Villena F., Wang W., McMillan L., Threadgill D.W. (2007). Mamm. Genome.

[49] Brown S.D., Hardisty R.E. (2003). Semin. Cell Dev. Biol..

[50] Chen Y., Zhu J., Lum P.Y., Yang X., Pinto S., MacNeil D.J., Zhang C., Lamb J., Edwards S., Sieberts S.K. (2008). Nature.

[51] Schadt E.E., Lamb J., Yang X., Zhu J., Edwards S., Guhathakurta D., Sieberts S.K., Monks S., Reitman M., Zhang C. (2005). Nat. Genet..

[52] Falconer D.S., Mackay T.F.C. (1996). Quantitative Genetics.

[53] Hanson W.D. (1959). Genetics.

[54] Hanson W.D. (1959). Genetics.

[55] Yu X., Bauer K., Wernhoff P., Koczan D., Moller S., Thiesen H.J., Ibrahim S.M. (2006). J. Immunol..

[56] Zhang S., Lou Y., Amstein T.M., Anyango M., Mohibullah N., Osoti A., Stancliffe D., King R., Iraqi F., Gershenfeld H.K. (2005). Mamm. Genome.

[57] Wang M., Lemon W.J., Liu G., Wang Y., Iraqi F.A., Malkinson A.M., You M. (2003). Cancer Res..

[58] Wang X., Le Roy I., Nicodeme E., Li R., Wagner R., Petros C., Churchill G.A., Harris S., Darvasi A., Kirilovsky J. (2003). Genome Res..

[59] Iraqi F., Clapcott S.J., Kumari P., Haley C.S., Kemp S.J., Teale A.J. (2000). Mamm. Genome.

[60] Kang H.M., Zaitlen N.A., Wade C.M., Kirby A., Heckerman D., Daly M.J., Eskin E. (2008). Genetics.

[61] Valdar W., Holmes C.C., Mott R., Flint J. (2009). Genetics.

[62] Valdar W., Solberg L.C., Gauguier D., Burnett S., Klenerman P., Cookson W.O., Taylor M.S., Rawlins J.N., Mott R., Flint J. (2006). Nat. Genet..

[63] Crow J.F. (1986). Basic Concepts in Population, Quantitative and Evolutionary Genetics.

[64] McClearn G.E., Wilson J.R., Meredith W., Lindzey G., Thiessen D. (1970). The use of isogenic and heterogenic mouse stocks in behavioral research. Contributions to Behavior-Genetic Analysis: The Mouse as a Prototype.

[65] Demarest K., McCaughran J., Mahjubi E., Cipp L., Hitzemann R. (1999). J. Neurosci..

[66] Mott R., Talbot C.J., Turri M.G., Collins A.C., Flint J. (2000). Proc. Natl Acad. Sci. USA.

[67] Shifman S., Bell J.T., Copley R.R., Taylor M.S., Williams R.W., Mott R., Flint J. (2006). PLoS Biol..

[68] Hitzemann R., Malmanger B., Cooper S., Coulombe S., Reed C., Demarest K., Koyner J., Cipp L., Flint J., Talbot C. (2002). Genes Brain Behav..

[69] Talbot C.J., Radcliffe R.A., Fullerton J., Hitzemann R., Wehner J.M., Flint J. (2003). Mamm. Genome.

[70] Talbot C.J., Nicod A., Cherny S.S., Fulker D.W., Collins A.C., Flint J. (1999). Nat. Genet..

[71] Guenet J.L., Bonhomme F. (2003). Trends Genet..

[72] Laurie C.C., Nickerson D.A., Anderson A.D., Weir B.S., Livingston R.J., Dean M.D., Smith K.L., Schadt E.E., Nachman M.W. (2007). PLoS Genet..

[73] Purcell S., Neale B., Todd-Brown K., Thomas L., Ferreira M.A., Bender D., Maller J., Sklar P., de Bakker P.I., Daly M.J. (2007). Am. J. Hum. Genet..

[74] Donnelly P. (2008). Nature.

[75] Ghazalpour A., Doss S., Kang H., Farber C., Wen P.Z., Brozell A., Castellanos R., Eskin E., Smith D.J., Drake T.A. (2008). PLoS Genet..

[76] Manenti G., Galbiati F., Noci S., Dragani T.A. (2003). Carcinogenesis.

[77] Yalcin B., Willis-Owen S.A., Fullerton J., Meesaq A., Deacon R.M., Rawlins J.N., Copley R.R., Morris A.P., Flint J., Mott R. (2004). Nat. Genet..

[78] Lynch C.J. (1969). Lab Anim. Care.

[79] Ferris S.D., Sage R.D., Wilson A.C. (1982). Nature.

[80] Beck J.A., Lloyd S., Hafezparast M., Lennon-Pierce M., Eppig J.T., Festing M.F., Fisher E.M. (2000). Nat. Genet..

[81] Bonhomme F. (1986). Curr. Top. Microbiol. Immunol..

[82] Wang X., Korstanje R., Higgins D., Paigen B. (2004). Genome Res..

[83] Manenti G., Galbiati F., Gianni-Barrera R., Pettinicchio A., Acevedo A., Dragani T.A. (2004). Oncogene.

[84] Park Y.G., Clifford R., Buetow K.H., Hunter K.W. (2003). Genome Res..

[85] Liao G., Wang J., Guo J., Allard J., Cheng J., Ng A., Shafer S., Puech A., McPherson J.D., Foernzler D. (2004). Science.

[86] Grupe A., Germer S., Usuka J., Aud D., Belknap J.K., Klein R.F., Ahluwalia M.K., Higuchi R., Peltz G. (2001). Science.

[87] Wade C.M., Kulbokas E.J., Kirby A.W., Zody M.C., Mullikin J.C., Lander E.S., Lindblad-Toh K., Daly M.J. (2002). Nature.

[88] Lindblad-Toh K., Winchester E., Daly M.J., Wang D.G., Hirschhorn J.N., Laviolette J.P., Ardlie K., Reich D.E., Robinson E., Sklar P. (2000). Nat. Genet..

[89] Wiltshire T., Pletcher M.T., Batalov S., Barnes S.W., Tarantino L.M., Cooke M.P., Wu H., Smylie K., Santrosyan A., Copeland N.G. (2003). Proc. Natl Acad. Sci. USA.

[90] Frazer K.A., Wade C.M., Hinds D.A., Patil N., Cox D.R., Daly M.J. (2004). Genome Res..

[91] Ideraabdullah F.Y., de la Casa-Esperon E., Bell T.A., Detwiler D.A., Magnuson T., Sapienza C., de Villena F.P. (2004). Genome Res..

[92] Yalcin B., Fullerton J., Miller S., Keays D.A., Brady S., Bhomra A., Jefferson A., Volpi E., Copley R.R., Flint J. (2004). Proc. Natl Acad. Sci. USA.

[93] Frazer K.A., Eskin E., Kang H.M., Bogue M.A., Hinds D.A., Beilharz E.J., Gupta R.V., Montgomery J., Morenzoni M.M., Nilsen G.B. (2007). Nature.

[94] Yang H., Bell T.A., Churchill G.A., Pardo-Manuel de Villena F. (2007). Nat. Genet..

[95] Payseur B.A., Place M. (2007). Genetics.

[96] Manenti G., Galvan A., Pettinicchio A., Trincucci G., Spada E., Zolin A., Milani S., Gonzalez-Neira A., Dragani T.A. (2009). PLoS Genet..

[97] Kirby A., Kang H.M., Wade C.M., Cotsapas C., Kostem E., Han B., Furlotte N., Kang E.Y., Rivas M., Bogue M. (2010). Genetics.

[98] Liu P., Vikis H., Lu Y., Wang D., You M. (2007). PLoS ONE.

[99] Liu P., Wang Y., Vikis H., Maciag A., Wang D., Lu Y., Liu Y., You M. (2006). Nat. Genet..

[100] Pletcher M.T., McClurg P., Batalov S., Su A.I., Barnes S.W., Lagler E., Korstanje R., Wang X., Nusskern D., Bogue M.A. (2004). PLoS Biol..

[101] Bennett B.J., Farber C.R., Orozco L., Kang H.M., Ghazalpour A., Siemers N., Neubauer M., Neuhaus I., Yordanova R., Guan B. (2010). Genome Res..

[102] Flint J., Mackay T.F. (2009). Genome Res..

[103] Yang X., Deignan J.L., Qi H., Zhu J., Qian S., Zhong J., Torosyan G., Majid S., Falkard B., Kleinhanz R.R. (2009). Nat. Genet..

[104] Wilkie A.O. (2005). Cytokine Growth Factor Rev..

[105] Flint J., Mott R. (2008). Nature.

[106] Long A.D., Mullaney S.L., Mackay T.F.C., Langley C.H. (1996). Genetics.

[107] Darvasi A. (2005). Trends Genet..

[108] Schadt E.E. (2009). Nature.

[109] Flint J., Mott R. (2001). Nat. Rev. Genet..

[110] Valdar W., Solberg L.C., Gauguier D., Cookson W.O., Rawlins J.N., Mott R., Flint J. (2006). Genetics.

